# Multiscale spatiotemporal heterogeneity of zeolite-catalyzed methanol-to-hydrocarbons reaction

**DOI:** 10.1093/nsr/nwag255

**Published:** 2026-05-08

**Authors:** Jinxi Hou, Yiyao Chen, Yiming Liu, Yuchun Zhi, Yingxu Wei, Zhongmin Liu

**Affiliations:** Dalian National Laboratory for Clean Energy, National Engineering Research Center of Lower-Carbon Catalysis Technology, Dalian Institute of Chemical Physics, Chinese Academy of Sciences, Dalian 116023, China; Energy College, University of Chinese Academy of Sciences, Beijing 100049, China; Dalian National Laboratory for Clean Energy, National Engineering Research Center of Lower-Carbon Catalysis Technology, Dalian Institute of Chemical Physics, Chinese Academy of Sciences, Dalian 116023, China; Energy College, University of Chinese Academy of Sciences, Beijing 100049, China; Dalian National Laboratory for Clean Energy, National Engineering Research Center of Lower-Carbon Catalysis Technology, Dalian Institute of Chemical Physics, Chinese Academy of Sciences, Dalian 116023, China; Energy College, University of Chinese Academy of Sciences, Beijing 100049, China; Dalian National Laboratory for Clean Energy, National Engineering Research Center of Lower-Carbon Catalysis Technology, Dalian Institute of Chemical Physics, Chinese Academy of Sciences, Dalian 116023, China; State Key Laboratory of Catalysis, Dalian Institute of Chemical Physics, Chinese Academy of Sciences, Dalian 116023, China; Energy College, University of Chinese Academy of Sciences, Beijing 100049, China; Dalian National Laboratory for Clean Energy, National Engineering Research Center of Lower-Carbon Catalysis Technology, Dalian Institute of Chemical Physics, Chinese Academy of Sciences, Dalian 116023, China; State Key Laboratory of Catalysis, Dalian Institute of Chemical Physics, Chinese Academy of Sciences, Dalian 116023, China; Energy College, University of Chinese Academy of Sciences, Beijing 100049, China; Dalian National Laboratory for Clean Energy, National Engineering Research Center of Lower-Carbon Catalysis Technology, Dalian Institute of Chemical Physics, Chinese Academy of Sciences, Dalian 116023, China; State Key Laboratory of Catalysis, Dalian Institute of Chemical Physics, Chinese Academy of Sciences, Dalian 116023, China; Energy College, University of Chinese Academy of Sciences, Beijing 100049, China

**Keywords:** MTH, zeolite, diffusion, multiscale, spatiotemporal heterogeneity

## Abstract

The methanol-to-hydrocarbons (MTH) process provides a sustainable route to light olefins, aromatics, and gasoline-range products with methanol synthesized via syngas platform molecules obtained from nonpetroleum alternative feedstocks, such as coal, biomass, and natural gas. Zeolite-catalyzed MTH reactions inherently exhibit multiscale heterogeneity, which is intrinsically linked to the rational design of high-efficiency industrial catalysts and the regulation of product selectivity. Despite extensive research on zeolite-based MTH catalysis, most studies focused on a single scale and a multiscale understanding that connects molecular-scale, crystal-scale, particle-scale, and reactor-scale performance remains elusive. This review integrates recent advances in spectroscopic characterization, molecular imaging, and spatially resolved techniques to dissect the heterogeneity of the MTH reaction (i.e. from molecular diffusion to acidity, temperature distribution, and coke deposition) across four levels and to clarify how these nonuniform microenvironments emerge, interact, and propagate. By integrating insights across these four scales, we aim to bridge micro- (molecule) and macro-processes (reactor), summarize both the adverse and favorable effects of multiscale heterogeneity in zeolite‑catalyzed processes, reveal spatiotemporal heterogeneity as the intrinsic and crucial principle of MTH reaction over zeolites, and expect this knowledge to provide theoretical support for the rational design of new-generation high-efficiency catalysts, enabling a more effective and selective MTH process.

## Teaser

This review summarizes multiscale spatiotemporal heterogeneities in zeolite-catalyzed methanol-to-hydrocarbon reactions across molecular, crystal, particle, and reactor scales, offering key insights for high-efficiency catalyst design and reaction regulation.

## INTRODUCTION

The methanol-to-hydrocarbons (MTH) process can be classified according to its main products, such as methanol-to-olefins (MTO), methanol-to-aromatics (MTA), and methanol-to-gasoline [1,2]. Starting from coal, natural gas, CO_2_, or biomass—via syngas to methanol and then to light olefins, this process bridges the gap between petrochemicals, coal chemicals, and natural gas chemicals [3]. After nearly three decades of exploration, the Dalian Institute of Chemical Physics of the Chinese Academy of Sciences successfully achieved the industrialization of the MTO technology in 2010. This technology has been continuously innovated and the third-generation DMTO-Ⅲ process has now been developed [4]. However, the continuous upgrading of industrial technology requires a deeper and more comprehensive understanding of methanol conversion process and mechanism.

The MTO reaction takes place within the confined channels of microporous molecular sieves and is driven by a complex autocatalytic process. The active intermediate species remaining on the catalyst surface continuously evolve during the reaction, resulting in a dynamic characteristic of methanol conversion. The methanol conversion reaction network gradually evolves and becomes more complex as the reaction proceeds [1,2,5–7]. The entire MTO reaction process can be divided into three stages. During the induction period, methanol undergoes a dehydration process, thereby generating surface methoxy species and initial C–C bond-containing intermediates [8,9]. In the stage of highly efficient methanol conversion, olefins, methylcyclopentenyl species, and aromatic species serve as active hydrocarbon pool species. They not only independently guide their respective catalytic cycles (olefins-based, cyclopentadienyl-based, and aromatics-based cycle), but also mutually couple with each other to jointly construct a highly active and complex supercycle reaction network, driving the efficient conversion of methanol into olefins [6,10,11]. In the deactivation stage, reaction-active intermediate species gradually transform into condensed aromatic coke deposition species through alkylation, ring formation, hydrogen transfer, etc., further increasing the complexity of the methanol reaction system and ultimately leading to the coke deposition and deactivation of zeolite [12–14]. After decades of research, substantial progress has been made in understanding the MTH reaction mechanism. However, as research has deepened, there is a growing recognition of the complexity of the MTO reaction mechanism and the understanding of its complex reaction route and network remains far from comprehensive. Moreover, zeolite-catalyzed MTH reactions inherently exhibit multiscale heterogeneity, and the spatiotemporal heterogeneities occurring during the MTH process further add to the complexity of the reaction. These spatiotemporal heterogeneities—arising from molecular diffusion to acidity, temperature distribution, and coke deposition—govern the dynamic interplay between diffusion, reaction, and deactivation, which leads to clear-cut distinctions in product selectivity and catalyst lifetime. Therefore, it is important to systematically investigate the spatiotemporal heterogeneities of MTH reaction processes at different scales. Elucidating the dynamic evolution of the MTH reaction and the spatiotemporal coupling mechanisms among diffusion, reaction, and deactivation is not only crucial for the rational design of new-generation high-efficiency catalysts but also offers valuable insights for a wide range of other zeolite-catalyzed processes, such as fluid catalytic cracking (FCC), aromatic cracking, oil upgrading, CO₂ hydrogenation, syngas conversion, etc.

**Figure 1.**
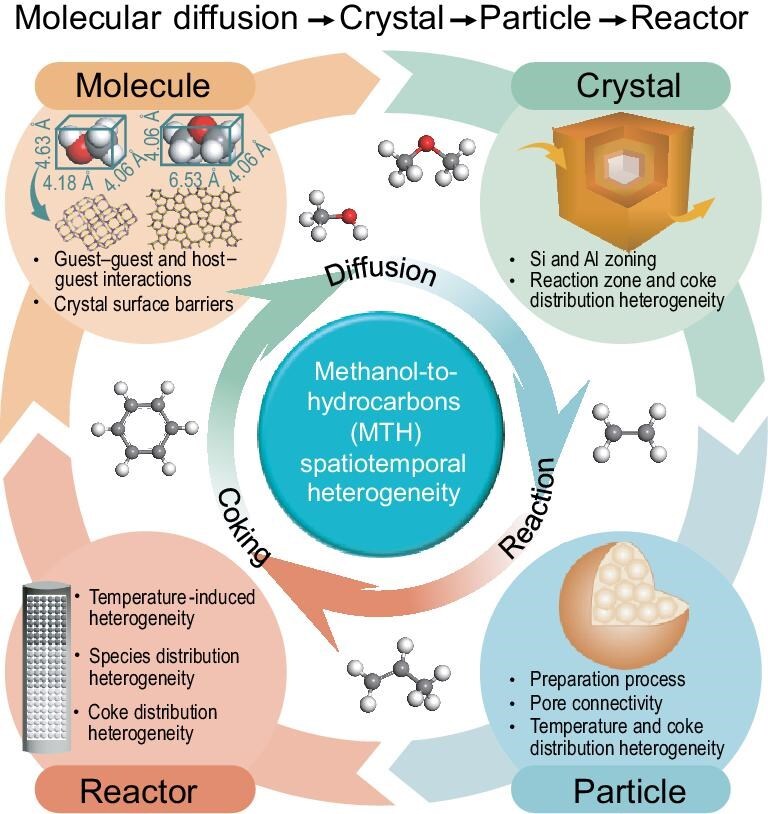
Multiscale spatiotemporal heterogeneity in the MTH reaction.

While there have been several excellent review articles in the field of MTH reaction, most studies focused on a single scale and only a small portion of reviews dealt with the spatiotemporal heterogeneity [2,5,15,16]. A review with comprehensive analysis of spatiotemporal heterogeneity from a multiscale viewpoint is still lacking. In this review, we emphasize elucidating the spatiotemporal heterogeneity of the MTH reaction across the molecular diffusion, crystal, particle, and reactor scales (Fig. 1). We first dissect diffusion heterogeneity, summarizing how guest–guest and host–guest interactions, zeolite topology, acid site microenvironments, crystal surface barriers, and confinement-induced anomalous transport effects cooperate to affect or modulate mass transfer. Next, we examine heterogeneity within crystals, where heterogeneous acid distribution, surface diffusion barriers, and the higher near-surface species concentration collectively result in surface-to-core decreasing deactivation gradients. At the particle scale, we highlight how the dispersion of zeolite components, binder properties, and pore connectivity affect mass transfer, which in turn influences coke distribution. Finally, at the reactor scale, we analyse temperature gradients and moving reaction fronts that drive axial and radial heterogeneities in product and coke profiles. By organizing the literature across these four scales, we aim to refine insights into the spatiotemporal heterogeneities of MTH reaction processes across the molecular diffusion, crystal, particle, and reactor scales and expect to provide guidance for the rational design of MTH catalysts and other zeolite‑catalyzed processes.

## DIFFUSION HETEROGENEITY IN THE MTH REACTION

Diffusion heterogeneity is a fundamental source of spatial nonuniformity in the MTH reaction, arising from the dynamic interplay among molecular identity, framework topology, acid sites, and surface transport resistance. At the molecular level, differences in size, polarity, and confinement lead to direction-dependent diffusivities, while guest–guest interactions and the progressive formation of hydrocarbon-pool species create evolving diffusion landscapes. Within the zeolite channel, topological constraints and acid sites distribution further amplify these variations. Additionally, surface barriers from defects and compositional inhomogeneities introduce extra resistance, decoupling bulk diffusion from effective transport. Collectively, these multiscale effects drive pronounced diffusion heterogeneity, which governs the emergence and propagation of spatiotemporal nonuniformities during the MTH process.

### Guest molecule diffusion heterogeneity

**Figure 2.**
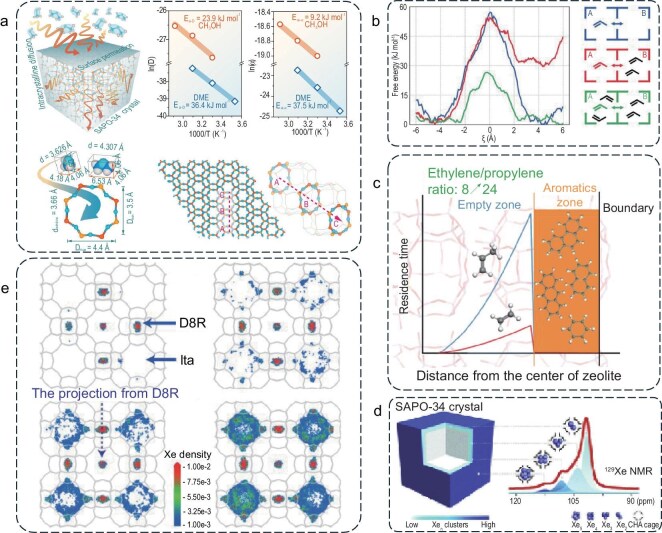
Guest molecule diffusion heterogeneity in the MTH reaction. (a) Diffusion behavior of methanol and dimethyl ether (DME) in SAPO-34, including intracrystalline diffusivity, surface permeability, molecular size effects, and CHA intercage diffusion pathways. Adapted from ref. [7] with permission of the Oxford University Press. (b) Free-energy profiles for propene diffusion across an 8-MR in SAPO-34 at 600 K under varying cage propene loadings from Ab initio umbrella sampling (AI-US) simulations. Adapted from ref. [17] with permission of the Royal Society of Chemistry. (c) Nonuniform mesoscale distributions of aromatic species in zeolite cages modulate olefin diffusion and residence time, leading to diffusion-improved ethylene selectivity in MTO catalysis. Adapted from ref. [20] with permission of the American Chemical Society. (d) Possible spatial distribution of xenon clusters within a single SAPO-34 crystal, where intensity indicates a decreasing number of xenon atoms per cage from the surface toward the interior. ^129^Xe magic angle spinning (MAS) NMR spectra were collected at a spinning rate of 8 kHz. Adapted from ref. [21] with permission of the American Chemical Society. (e) Density maps of xenon adsorbed in RHO at 298 K with increased loading 1, 3, 6 and 11 atoms per unit cell. The crystal structure of RHO is viewed along the [001] direction. Adapted from ref. [22] with permission of the American Chemical Society

Diffusion heterogeneity in the MTH reaction already manifests at the level of reactant molecules within microporous zeolite catalysts. In CHA-type frameworks such as SAPO-34, methanol and DME exhibit distinct transport behaviors (Fig. 2a). Multiscale analyses showed that methanol reaction proceeds in a layer-by-layer manner and exhibits a heterogeneous reaction and deactivation mode, while DME experiences stronger diffusion constraints than methanol, leading to attenuated local enrichment and longer and homogeneous reaction zones within individual crystals [7]. Such reactant-specific diffusion disparity establishes an early spatiotemporal heterogeneity in the effective concentration field, which precedes and modulates downstream hydrocarbon formation.

As the reaction progresses toward hydrocarbon products, intrinsic molecular size and shape further affect diffusion pathways and rates. Molecular simulations resolving intercage hopping across CHA eight-membered ring (8-MR) (Fig. 2b) demonstrated that olefin diffusion is an activated process with a pronounced size dependence, where ethene diffuses more readily than propene due to lower steric hindrance and reduced energy barriers [17]. For diffusion through acid-free 8-MR at 450 K, the calculated free-energy barriers are 21.2 kJ·mol^−1^ for ethylene and 40.9 kJ·mol^−1^ for propylene, directly quantifying the stronger transport penalty for the larger olefin [17]. These simulation results are corroborated experimentally by pulsed-field gradient (PFG) nuclear magnetic resonance (NMR) measurements, which revealed higher self-diffusion coefficients for ethene compared to ethane (at 295 K and time on stream (TOS) = 0 min, the self-diffusion coefficients of ethane and ethene in the fresh large-crystal SAPO-34 sample were 6.7 × 10^−12^ and 1.3 × 10^−11^ m^2^·s^−1^, respectively) and an increasing diffusion selectivity toward smaller molecules under reaction conditions [18]. Together, these results indicate that even among light hydrocarbons, diffusion is intrinsically nonuniform and preferentially restricts larger guest molecules.

Crucially, coke formation further disturbs the intrinsic size-dependent diffusion, amplifying spatiotemporal heterogeneity as deactivation proceeds. Coke deposition decreases the diffusion coefficients and increases activation energies, disproportionately affecting larger molecules [19]. Using CH_4_ and C_2_H_4_ as probe molecules at a loading of 0.5 molecules per CHA cage at 298 K, Gao *et al.* [19] showed that the self-diffusion coefficient of methane decreased from 4.11 × 10^−11^ m^2^·s^−1^ in the fresh catalyst to 2.71 × 10^−11^ m^2^·s^−1^ at 18 min and 3.08 × 10^−12^ m^2^·s^−1^ at 49 min (the coke amount reaches 13.9 wt%). Besides, retained aromatics and coke species narrow or partially block zeolite pores, further intensifying the heterogeneous diffusion and the diffusion limitation of bulky molecules (Fig. 2c). Mesoscale analyses further suggest that such coke-induced diffusion resistance propagates nonuniformly from the crystal exterior toward the interior, thereby reinforcing spatial gradients in hydrocarbon residence time and diffusion selectivity [20].

Overall, diffusion heterogeneity in MTH is an intrinsic and dynamically evolving property of zeolite catalysts. It stems from the inherent molecular-size effect among reactants and products and is progressively amplified by coke deposition. This heterogeneity affects hydrocarbon-pool evolution, biases product selectivity toward smaller molecules, promotes spatially nonuniform coke deposition, and reduces catalyst utilization efficiency.

### Diffusion heterogeneity induced by the topological structure of zeolite

Topology-induced diffusion heterogeneity in the MTH reaction becomes evident when comparing cage-type and channel-type zeolites, where pore dimensions, connectivity, and adsorption environments regulate mass transport. In cage-based frameworks like CHA and RHO, diffusion is governed by window-mediated intercage exchange, yet the specific topology dictates how nonuniformity develops. In CHA-type zeolites (e.g. SAPO-34), direct experimental probing revealed pronounced spatial heterogeneity in adsorption and diffusion within single crystals (Fig. 2d), arising from the confinement imposed by 8-MR windows that connect large cages and limit intercage transport [21]. By contrast, in the RHO framework, diffusion heterogeneity is strongly coupled to topology-defined preferential adsorption sites (Fig. 2e). Xenon adsorption studies demonstrated that double eight-membered rings act as dominant adsorption centers and their progressive occupation leads to loading-dependent mass-transfer limitations [22].

Moving to channel-type zeolites, diffusion heterogeneity becomes even more topology-selective. A representative comparison between the one-dimensional frameworks ZSM-12 (MTW, 5.6 × 6.0 Å) and ZSM-22 (TON, 4.6 × 5.7 Å) showed that small variations in pore size translate into substantial differences in diffusivity and, in turn, catalytic performance. Molecular dynamics simulations revealed that methanol and olefins diffuse several times faster in ZSM-12 than in the narrower ZSM-22 [23]. The active polymethylbenzene species are easily formed in ZSM-12, thereby promoting the aromatic-based cycle and enhancing ethylene production. While in the narrower channels of ZSM-22, the polymethylbenzene species are easily ‘stuck’ and lead to deactivation. In this sense, topology-controlled diffusion not merely affects mass transport rates but also actively regulates the spatiotemporal distribution of reactive intermediates and reaction pathways inside the crystal [23].

**Figure 3.**
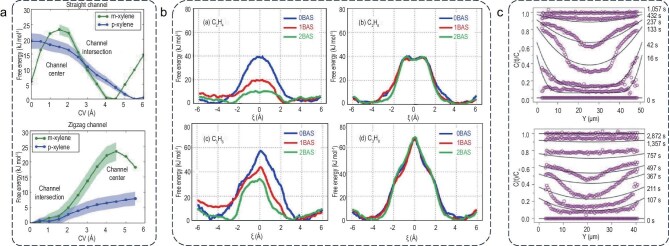
Diffusion heterogeneity induced by zeolite topology, acid sites, and crystal surface barriers. (a) Free-energy profiles of p-xylene and m-xylene diffusion in straight and zig-zag channels from blue-moon simulations, including standard deviations. Adapted from ref. [26] with permission of the Springer Nature. (b) Free-energy profiles for ethene, ethane, propene, and propane diffusion at 600 K through an eight-ring window of SAPO-34 containing 0, 1, or 2 Br∅nsted acid sites (BASs). Adapted from ref. [27] with permission of the Wiley-VCH GmbH. (c) Interference microscopy (IFM)-measured intracrystalline methanol concentrations in two similar SAPO-34 crystals at 298 K after a 0–1 mbar pressure step, with one dimensional (1D) fitted profiles. Adapted from ref. [28] with permission of the Springer Nature.

Even within a single framework, topology can generate intrinsic diffusion nonuniformity, as exemplified by ZSM-5 zeolite. The coexistence of straight and sinusoidal channels gives rise to pronounced diffusion anisotropy, with guest molecules exhibiting channel-dependent mobilities and heterogeneous diffusion trajectories (Fig. 3a). Single-molecule tracking showed diffusion coefficients spanning several orders of magnitude, with a mean diffusion coefficient of 2.64 ± 0.24 × 10^−14^ m^2^·s^−1^ in straight channels, nearly twice that in sinusoidal channels (1.38 ± 0.11 × 10^−14^ m^2^·s^−1^) [24]. It showed that channel geometry alone can induce strong transport heterogeneity in otherwise chemically uniform materials [24]. In MFI, the diffusion of olefins exhibited temperature dependence. At low temperatures, propylene tends to diffuse along the zig-zag channels and assumes a morphological state parallel to the horizontal plane. In contrast, at high temperatures, propylene tends to adopt a vertical morphology, which facilitates diffusion along the straight channels and consequently results in a higher diffusion rate [24–26]. Radial distribution function analysis further confirmed that increasing temperature weakens the interaction between propylene and Brønsted acid sites, thereby reducing adsorption strength and facilitating faster molecular diffusion [25].

In summary, zeolite topology affects diffusion heterogeneity by defining pore dimensionality, window aperture, and channel connectivity. Cage, channel, and anisotropic frameworks inherently impose distinct transport constraints and direction-dependent mobilities. Diffusion heterogeneity thus directly arises from framework geometry, making topology a primary determinant of mass transport behavior.

### Diffusion heterogeneity affected by acid sites in zeolite

In the MTH reaction over SAPO-34, diffusion heterogeneity of olefins is alleviated by Brønsted acid sites located at the 8-MR windows, where guest–acid interactions directly modulate intercage transport. Molecular simulations showed that Brønsted acid sites located on the 8-MR can significantly lower the free-energy barriers for diffusion by stabilizing transient π–H complexes between the olefin and the acidic proton [17]. For instance, at 450 K the free-energy barrier for ethene diffusion decreases from about 30 kJ·mol^−1^ in an acid-free 8-MR to about 15 kJ·mol^−1^ when acid sites are present on the ring [17]. Consequently, acid-decorated windows act as preferential diffusion pathways, whereas acid-free windows impose higher barriers and serve as local transport bottlenecks.

This promotional effect is strongly molecule-specific. Combined first-principles simulations, PFG NMR and temporal analysis of products (TAP) experiments demonstrated that alkene diffusivities increase with increasing acid site density on the 8-MR, while alkane diffusion remains essentially unaffected by acidity [17]. For ethene and propene, diffusion barriers are substantially reduced when one or two Brønsted acid sites decorate the window, whereas ethane and propane exhibit nearly constant barriers regardless of acid site presence (Fig. 3b). Moreover, asymmetric placement of a single acid site on the 8-MR can introduce direction-dependent diffusion barriers, further enhancing spatial nonuniformity at the molecular scale [27].

Brønsted acid sites on the 8-MR of SAPO-34 act as localized diffusion promoters for olefins, selectively facilitating their transport through an otherwise strongly hindered pore system. As acid sites are nonuniformly distributed among the multiple cages, alkene diffusion becomes heterogeneous, creating diverse local reaction environments within the crystal and providing a direct structural origin for spatiotemporal nonuniformity in the MTH process.

### Diffusion heterogeneity induced by crystal surface barriers

Surface barriers are an additional resistance at the external surface of zeolite crystals, which represent an important source of diffusion nonuniformity in the MTH process. Early phenomenological studies already recognized surface barriers as species-dependent permeation resistances arising from structural or chemical modifications within a thin surface layer [28,29]. Recent advances (Fig. 3c) have directly visualized that guest uptake can be limited by surface permeation and that substantial intercrystal and intracrystal heterogeneity in surface barriers exist even among nominally identical nanoporous crystals [28,30]. In SAPO-34, quantitative transport analyses further demonstrated that overall mass transfer can be dominated by surface barriers, whose magnitude varies strongly with external surface properties such as acidity, highlighting the inherently nonuniform nature of surface-controlled diffusion [31].

The surface barriers are governed by surface structure and chemistry. Surface modification or defect-related narrowing of pore entrances can markedly increase surface resistance, whereas treatments that remove surface obstructions or open pore mouths reduce the barrier with little effect on the crystal interior. For example, controlled surface deposition and mild acid etching of SAPO-34 selectively increase or decrease surface permeability, respectively, allowing surface barriers to be tuned independently of intracrystalline diffusion [32]. At the molecular level, terminal groups at the external surface provided a clear mechanistic basis. Isolated silanol species could both enhance guest–surface interactions and locally constrain pore entrances, thereby lowering surface permeability and generating asymmetric uptake and release behavior [33]. In addition, environmental exposure, such as humidity, preferentially degrades the external surface, causing a rapid increase in surface resistance while bulk diffusivity remains relatively less affected [31].

Systematic control of surface permeability in SAPO-34 has been shown to directly influence catalytic lifetime and product selectivity. Reduced surface barriers facilitate product removal (the maximum selectivity toward light olefins is 81.6%) and prolong catalyst lifetime, whereas enhanced barriers hinder outward diffusion, promote secondary reactions inside the cages (the maximum selectivity toward light olefins is 73.6%), and accelerate deactivation [32]. Overall, zeolite surface barriers represent a controllable, surface-localized origin of diffusion nonuniformity whose causes, governing factors, and catalytic consequences are mutually entangled.

### Diffusion heterogeneity caused by confinement-induced anomalous transport effects

In addition to conventional diffusion behaviors, zeolites employed in the MTH reaction feature typical confined pore channels, where anomalous phenomena may arise depending on factors such as pore architecture, molecular structure, temperature, and loading, all of which can influence catalytic performance [34,35]. For instance, the thermal resistance effect [36] demonstrated that under confinement, elevated temperatures induce bending in long-chain molecules, increase diffusion resistance, and effectively mimic bulkier configurations—a process that may accelerate coke formation during MTH. Conversely, hyperloop-like diffusion [37] showed that in narrowly confined one-dimensional channels, long-chain molecules can achieve ultrafast transport by maintaining linearity and moving along the channel center, suggesting that appropriately sized channels can facilitate rapid product egress and mitigate catalyst deactivation. More recently, the molecular self-gating effect [38] revealed that under confinement, increasing concentration first induces a ‘traffic jam’ that hinders diffusion, but beyond a critical loading, molecular synergy enables a transition to ‘smooth traffic’ thereby alleviating diffusion limitations. This concentration-dependent behavior offers new insights into managing diffusion and coke suppression in MTH catalysis. Collectively, these studies underscore that diffusion behavior in confined spaces is governed by a delicate balance among molecular flexibility, pore architecture, temperature, and loading.

In summary, diffusion heterogeneity in the MTH reaction originates from five interrelated aspects. First, intrinsic molecular properties (molecular size, shape, and polarity) lead to distinct diffusion resistances for reactants, intermediates, and products. Second, guest–guest and host–guest interactions, particularly those involving hydrocarbon-pool species, dynamically affect diffusion pathways and increase the diffusion heterogeneity by narrowing effective pore apertures. Third, zeolite topology and channel architecture impose direction-dependent transport constraints, giving rise to pronounced anisotropic diffusion and topology-controlled selectivity. Fourth, crystal surface barriers introduce an additional layer of transport resistance and allow for the decoupling of intracrystalline diffusion from the ensemble diffusion behavior. Finally, beyond these conventional factors, confinement itself can induce anomalous transport behaviors, including the thermal resistance effect, hyperloop-like diffusion, and the molecular self-gating effect, indicating that molecular transport in zeolites is not only spatially heterogeneous but also dynamically regulated by temperature, molecular configuration, and loading under reaction conditions. Together, these factors establish diffusion as a highly anisotropic and dynamically evolving process, providing the mechanistic foundation for spatiotemporal nonuniformity in activity, selectivity, and deactivation.

Importantly, diffusion heterogeneity does not remain a purely molecular phenomenon. When combined with nonuniform acid site distributions and compositional zoning inherited from crystallization, it manifests as crystal-scale heterogeneity, where reaction zones, coke accumulation, and deactivation fronts develop nonuniformly within zeolite crystals. This coupling between diffusion and intracrystalline chemical heterogeneity forms the basis of the crystal-scale spatiotemporal phenomena discussed in the following section.

## CRYSTAL-SCALE HETEROGENEITY IN THE MTH REACTION

Crystal-scale heterogeneity arises from structural, compositional, and dynamic gradients within individual zeolite crystals, all of which strongly dictate activity, selectivity, and deactivation behavior of MTH reaction. Synthesis inevitably imprints nonuniform Si and Al distributions, with core–shell architectures and defect-rich domains that predetermine local acidity and diffusion behavior. Under reaction conditions, temperature-dependent mobility of hydrocarbon-pool species reshapes intracrystalline reaction–diffusion coupling. Meanwhile, coke formation proceeds in a highly localized and framework-sensitive manner, generating distinct surface-to-core coke gradients. This section therefore examines crystal-level heterogeneity from the perspectives of synthesis-induced structural variation, temperature-driven reaction heterogeneity, and the spatiotemporal evolution of coke.

### Acid site distribution heterogeneity

**Figure 4.**
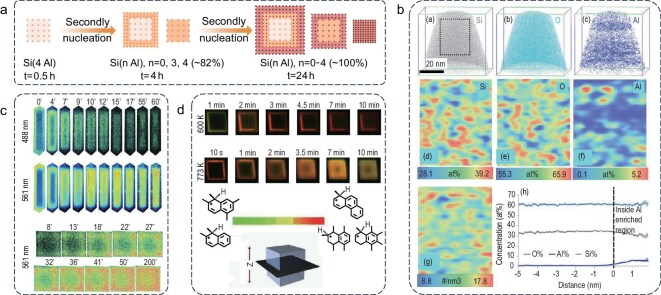
Heterogeneity in acid distribution, reaction zone distribution, and coke distribution within zeolite crystals. (a) Schematic illustration of crystal growth and silicon distribution. Adapted from ref. [40] with permission of the Elsevier. (b) APT results of fresh HZSM-5 reveal intrinsic periodic clustering of silicon, oxygen, and aluminum in the newly synthesized material. Adapted from ref. [44] with permission of the Springer Nature. (c) Confocal fluorescence intensity profiles of the HZSM-5 (upper) and H-SAPO-34 (lower) crystals during the MTO reaction at 660 K, as a function of time-on-stream. Adapted from ref. [48] with permission of the Wiley-VCH GmbH. (d) CFM images of a single 50 μm SAPO-34 crystal during the MTO reaction as a function of time-on-stream at 600 and 773 K. The fluorescence signal arises from the overlap of signals excited at λ = 488 and 561 nm, with the legend indicating the types of organic species associated with each excitation wavelength. Adapted from ref. [49] with permission of the Wiley-VCH GmbH.

Spatial heterogeneity of Brønsted acid sites within individual zeolite crystals is fundamentally rooted in synthesis-induced compositional zoning. For SAPO-34, crystallization studies showed that, rather than homogeneous substitution (Fig. 4a), Si is introduced through kinetically controlled processes such as island growth, leading to gradients in framework Si content across the crystal [39,40]. Because Brønsted acid sites in SAPO-34 are directly associated with isolated framework Si, this crystallization pathway inevitably imprints intracrystalline acid density heterogeneity, often manifested as acid-enriched outer regions relative to the crystal interior.

A conceptually similar yet mechanistically distinct origin of acid heterogeneity is observed in HZSM-5. Time-resolved crystallization studies indicated that ZSM-5 forms through a combination of nonclassical aggregation of Al-rich precursors and subsequent classical growth, during which aluminum is dynamically redistributed within the developing crystal [41]. This process commonly produces Al-enriched rims and Al-depleted cores, translating directly into spatially heterogeneous Brønsted acid sites [42].

The existence and catalytic relevance of this intracrystalline acid heterogeneity in HZSM-5 have been directly verified by spatially resolved experiments. Single-particle fluorescence microscopy revealed that in same batch of HZSM-5 zeolites, the Brønsted acid sites are nonuniformly distributed within the particles. Specifically, the particles can be classified into three categories: one type shows overall uniform high activity, another type has activity only in the peripheral regions while the interior is almost inactive, and the third type has no obvious activity overall. This discovery challenges the assumptions about the homogeneity of catalyst particles in traditional characterization methods [43]. Importantly, atom probe tomography (APT) provided direct, nanoscale evidence (Fig. 4b) that Al heterogeneity is an intrinsic structural feature of HZSM-5 rather than a reaction-formed artifact [44].

Despite differences in framework chemistry and crystallization mechanisms, both SAPO-34 and HZSM-5 exhibit synthesis-encoded intracrystalline acidity heterogeneity with acids enriched in the near-surface region of zeolite. This structural nonuniformity provides a fundamental microscopic origin for spatiotemporal heterogeneity in the MTH process.

### Reaction zone distribution heterogeneity

Spatially nonuniform reaction zones within individual zeolite crystals are closely related to reaction temperature. In HZSM-5, quasi-elastic neutron scattering combined with molecular dynamics simulations showed that methanol mobility increases markedly with temperature, transitioning from localized, adsorption-dominated dynamics at low temperatures to long-range intracrystalline diffusion at elevated temperatures [45]. At lower temperatures, methanol diffusion is sufficiently slow that conversion is confined near the external surface, whereas higher temperatures enhance methanol penetration into the crystal interior, increasing the volume fraction of the crystal that actively participated in the reaction [45].

This temperature-controlled diffusion behavior directly governs the spatial distribution of reaction zones within HZSM-5 crystals. Single-particle *operando* UV/Vis and confocal fluorescence microscopy (CFM) revealed that, under mild conditions, hydrocarbon pool species and subsequent carbonaceous products initially form near the crystal rim and gradually propagate inward with increasing temperature or time on stream (Fig. 4c), reflecting diffusion-limited access of methanol to internal acid sites [46–48]. At higher temperatures, accelerated methanol diffusion expands the active reaction zone but simultaneously promotes rapid formation of bulky aromatic species near pore mouths and external surfaces, which can in turn restrict access to the interior, leading to a dynamic redistribution of reaction zones over time. These observations highlight that reaction-zone heterogeneity in HZSM-5 is not static, but evolves from kinetically controlled, surface-localized reactivity to bulk diffusion-mediated spatiotemporal heterogeneity.

Similarly, SAPO-34 exhibits an analogous manifestation of reaction-zone heterogeneity due to its cage-based CHA topology. Single-particle microspectroscopy on large SAPO-34 crystals showed that, at lower temperatures, MTO reactions predominantly occur in an outer shell region (Fig. 4c and d), whereas higher temperatures enable deeper penetration and more homogeneous formation of hydrocarbon pool species throughout the crystal [48,49]. Compared to HZSM-5, the smaller cage windows of SAPO-34 impose stronger diffusion constraints, making the expansion of reaction zones with temperature more abrupt and sensitive to diffusion–reaction coupling.

These studies collectively demonstrate that intracrystalline reaction-zone heterogeneity in MTH is governed by the balance between temperature-dependent methanol diffusion and reaction kinetics. While both HZSM-5 and SAPO-34 display surface-biased reactivity, differences in pore topology modulate how diffusion controls the spatiotemporal evolution of active reaction zones within individual crystals.

### Coke distribution heterogeneity within zeolite crystals

Coke deposition within individual zeolite crystals is intrinsically spatially heterogeneous and its distribution strongly depends on framework topology, crystal size, reaction temperature, and diffusion behavior. For cage-based molecular sieves such as SAPO-34, available spectroscopic and microscopic studies consistently indicated that coke formation in SAPO-34 often tends to be enriched near the crystal periphery rather than homogeneously distributed throughout the crystal interior [19,50]. CFM, hyperpolarized xenon nuclear magnetic resonance (Xe NMR), and *operando* spectroscopic analyses demonstrated that bulky aromatic and polycyclic species preferentially accumulate within cages close to the external surface, forming a shell-like coke layer that progressively impedes inward diffusion of reactants and products [19,48]. This spatial confinement of coke is closely linked to the CHA topology, where large cages interconnected by narrow 8-MR windows favor local growth and retention of carbonaceous species once diffusion is hindered.

**Figure 5.**
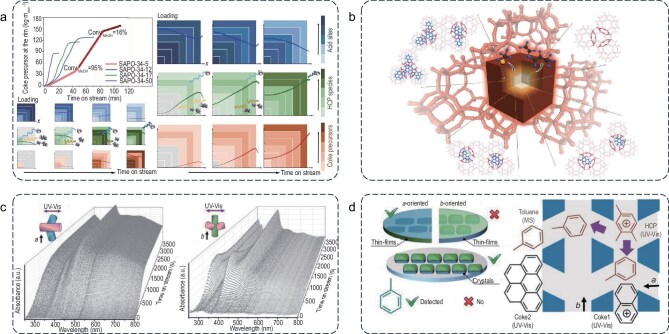
Coke distribution heterogeneity within zeolite crystals. (a) Schematic illustration of the spatiotemporal evolution of acid sites, hydrocarbon-pool species, and coke precursors during the MTO reaction on SAPO-34 zeolite crystals with different crystal sizes. Adapted from ref. [52] with permission of the Springer Nature. (b) Proposed comprehensive deactivation model highlighting the spatial distribution of structurally resolved coke species, ranging from confined active aromatics in the crystal interior to cross-linked, cage-spanning heavier polycyclic aromatic hydrocarbons (PAHs). Adapted from ref. [53] with permission of the Springer Nature. (c) *Operando* UV/Vis diffuse reflectance spectroscopy (DRS) spectra of HZSM-5 thin films with different orientations show that the *a*-oriented film (sinusoidal channels) exhibits a more pronounced absorption band at 620 nm (coke 2), whereas the *b*-oriented film (straight channels) displays a stronger absorption band at 420 nm (coke 1). Adapted from ref. [54] with permission of the Wiley-VCH GmbH. (d) Schematic of toluene effluents from *a*- and *b*-oriented ZSM-5 thin films and discrete crystals. Schematic representation of coke locations and aromatic formation during MTH derived from UV/Vis and on-line mass spectrometry (MS) results. Adapted from ref. [54] with permission of the Wiley-VCH GmbH.

The extent of such coke heterogeneity in SAPO-34 was further modulated by crystal size (Fig. 5a). Comparative studies on SAPO-34 crystals of different dimensions demonstrated that larger crystals exhibit more pronounced surface-enriched coke distributions, whereas smaller crystals tend to display a more uniform—but overall lower—coke content due to the shortened diffusion lengths and reduced residence times [19,51,52]. Molecular-level analyses further revealed that coke growth in cage-based zeolites proceeds via cross-linking of polycyclic aromatic hydrocarbons (Fig. 5b), which inherently promotes localized accumulation of coke within the crystal [53]. Thus, coke fronts in SAPO-34 advance from the surface toward the core, leading to persistent radial gradients in carbonaceous deposits.

In contrast, channel-based zeolites such as HZSM-5 exhibit distinct coke distribution patterns (Fig. 5c and d) governed by their interconnected channel architecture and crystallographic anisotropy [54]. Space- and time-resolved spectroscopic studies showed that coke formation in HZSM-5 initially concentrates near channel intersections and external crystal surfaces, followed by gradual penetration into the bulk along preferential diffusion directions [46,48]. APT and single-crystal spectroscopic analyses further demonstrated that coke clusters are spatially correlated with aluminum-rich regions and specific channel orientations, underscoring the coupling between framework heterogeneity and coke localization [44,50,55,56]. Reaction temperature exerts an additional level of control. Higher temperatures promote the formation of more condensed aromatic species and facilitate deeper coke penetration into the crystal, whereas lower temperatures favor surface-confined deposits that more rapidly induce diffusion limitations [48]. Importantly, recent atomic-resolution identification of coke molecular structures revealed that coke growth in HZSM-5 proceeds via well-defined molecular routes, involving successive alkylation–cyclization steps and dehydrogenative condensation mode between adjacent aromatic precursors across neighboring channel intersections [57]. This intersection-passing growth mechanism resembles the cage-passing coke growth of SAPO-34, underscoring the unity of coke growth inside zeolite pores.

At the crystal-scale, MTH heterogeneity originates from two coupled factors: nonuniform acid site distribution and diffusion limitations within crystals. Intrinsic compositional zoning inherited from crystallization creates spatially heterogeneous Brønsted acid sites, which govern where hydrocarbon-pool species preferentially form and evolve. Superimposed on this chemical heterogeneity, diffusion in microporous zeolites proceeds heterogeneously. Variations in intracrystalline diffusivity lead to nonuniform reactant penetration, localized accumulation of intermediates, and spatially resolved coke growth, ultimately producing nonuniform reaction zones and deactivation fronts within single crystals.

These intrinsic heterogeneities are further amplified when crystals are assembled with additives, supports, and binders into catalyst particles. At the particle scale, additional sources of heterogeneity arise from catalyst shaping, intracrystalline pore connectivity, and heat-/mass-transfer limitations within the particles. Therefore, understanding crystal-scale heterogeneity is essential for bridging insights obtained from single crystals and the performance of practical catalysts.

## PARTICLE-SCALE HETEROGENEITY IN THE MTH REACTION

MTH conversion exhibits pronounced spatiotemporal heterogeneity at the particle scale. Such heterogeneity governs where hydrocarbon-pool species first emerge, how coke fronts propagate, which regions deactivate prematurely, and how olefin selectivity develops over time. Recent advances have revealed that particle-scale heterogeneity is intrinsic to MTH catalysis and originates from differences in pore architecture, zeolite–binder interfaces, and heat-transfer limitations. Understanding these nonuniformities is essential for interpreting catalyst lifetime, optimizing reactor performance, and enabling the rational design of highly efficient MTH catalysts. This section primarily summarizes the structural heterogeneity introduced during particle preparation, the nonuniform connectivity of pore networks, and the heterogeneous spatial distribution of coke.

### Heterogeneity induced by the preparation process

In practical MTH catalysts, spatial heterogeneity is inevitably introduced during the preparation of zeolite-based catalyst bodies. One primary source of such heterogeneity arises from the dispersion degree of zeolite crystallites within the catalyst bodies. CFM and *operando* spectroscopic studies have demonstrated that poorly dispersed ZSM-5 aggregates form localized reaction hot spots, where intensified secondary reactions promote paraffin formation and coke accumulation, whereas a higher homogeneous dispersion of zeolite domains leads to more uniform utilization of acid sites and improved catalytic stability [58]. Thus, even with identical chemical composition, variations in spatial dispersion translate directly into nonuniform reaction zones inside catalyst particles.

Beyond dispersion, the zeolite loading further modulates this intraparticle heterogeneity. Increasing zeolite content enhances overall activity but simultaneously shortens intercrystalline distances, facilitating secondary reactions between neighboring domains and exacerbating local diffusion limitations. Imaging studies revealed that high loadings favor clustered reaction regions with accelerated deactivation, while lower loadings distribute activity more evenly throughout the catalyst bodies, albeit at the expense of volumetric productivity [58–60]. These findings highlight that zeolite loading does not simply scale with activity but reshapes the spatial reaction landscape within shaped catalysts.

The nature of the binder introduces an additional layer of heterogeneity. Rather than being inert diluents, common binders such as alumina, silica, and clays exhibit distinct catalytic and transport properties that influence local reaction environments [61–63]. Different binders were shown to induce binder-dependent variations in acid site density and speciation, macroporosity, mass-transfer characteristics, and product distribution, indicating that the binder does not act merely as an inert diluent, but creates a local environment with nonuniform chemical and transport properties [62,63]. Recent mechanistic studies showed that binder materials can actively participate in methanol conversion and hydrogen-transfer reactions, thereby modifying coke propensity and product distributions in the vicinity of zeolite domains [61]. Consequently, spatial variations in binder composition or binder–zeolite contact generate chemically heterogeneous regions within catalyst particles, reinforcing nonuniform reaction and deactivation patterns.

**Figure 6.**
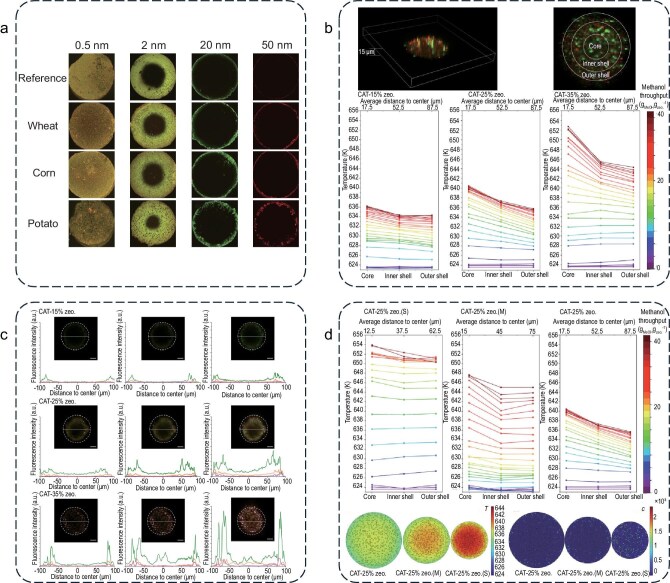
Preparation-induced and temperature-distribution heterogeneity at the particle scale. (a) Visualization of accessibility in shaped zeolite-based catalyst bodies using fluorescent nanoprobes (0.5, 2, 20, and 50 nm) after 24 h incubation, measured by CFM. Adapted from ref. [64] with permission of the American Chemical Society. (b) Confocal upconversion luminescence (UL) microscopy visualization of spatiotemporal temperature distributions in catalyst particles with different zeolite contents (15%, 25%, and 35%) during MTH, including 3D luminescence reconstruction, region-resolved analysis, and ratiometric temperature evolution. Adapted from ref. [65] with permission of the American Chemical Society. (c) Super-resolution CFM imaging of the spatiotemporal evolution of carbonaceous species in CAT-15%, CAT-25%, and CAT-35% zeolite-containing catalyst particles during MTH. Adapted from ref. [65] with permission of the American Chemical Society. (d) Spatiotemporal evolution of temperature rise within catalyst particles of different sizes by ratiometric thermometry using confocal UL microscopy during MTH reactions. Adapted from ref. [65] with permission of the American Chemical Society. 3D: three dimensional.

Finally, the incorporation of pore-forming agents during shaping provides a route to deliberately tune this heterogeneity (Fig. 6a). The addition of porogens such as starches creates meso- and macroporous networks that enhance pore connectivity and accessibility, mitigating sharp diffusion gradients from the particle exterior to the core. Advanced fluorescence imaging revealed that appropriately designed pore architectures enable deeper penetration of reactants and a more homogeneous distribution of hydrocarbon species during MTH conversion [59,64]. However, insufficient control over porogen size or distribution can conversely introduce new transport boundaries, underscoring the delicate balance between engineered and unintended heterogeneity.

Taken together, these studies demonstrate that spatial heterogeneity in catalyst particles is not solely an intrinsic property of zeolite crystals but is profoundly affected by particle preparation parameters, including dispersion, loading, binder choice, and pore-forming strategies. Understanding and controlling these preparation-induced heterogeneities is essential for bridging the gap between fundamental zeolite chemistry and the performance of industrially relevant MTH catalysts.

### Temperature distribution heterogeneity

In the MTH reaction, the strong exothermicity inherently creates nonuniform temperature fields within catalyst particles. Early studies demonstrated that even a single catalyst pellet exhibits pronounced internal and surface temperature gradients due to asymmetric heat and mass transfer conditions [66]. Such intraparticle temperature gradients are further amplified under kinetically fast reactions, where local heat generation outpaces heat dissipation, establishing hot zones that are nonuniformly distributed across the particle cross-section. These gradients imply that the reaction environment experienced by active sites is inherently position-dependent rather than uniform at the particle scale.

Beyond intrinsic heat generation, external operating conditions critically modulate the magnitude and symmetry of temperature heterogeneity. Variations in reactant gas flow rate alter local convective heat transfer coefficients, thereby altering surface temperature distributions and propagating asymmetric thermal profiles into the particle interior [66–68]. Moreover, the composition of the reaction atmosphere plays a decisive role in modulating temperature heterogeneity. Gases with lower thermal conductivities or different heat capacities can significantly shift the local catalyst temperature away from the reactor setpoint [69]. These effects highlight that temperature heterogeneity in MTH catalysts is not solely governed by particle properties, but emerges from a coupled interaction between reaction kinetics, heat transport, and reactor hydrodynamics.

Recent advances in nanoscale thermometry have fundamentally transformed the ability to directly probe these temperature fields. The development of luminescence-based nanothermometers, including lanthanide-doped nanoparticles and gallium-filled carbon nanotubes, has enabled noninvasive temperature measurements with submicrometer spatial resolution under catalytic conditions [67,69,70]. Building on these advances, *operando* luminescence thermometry combined with confocal microscopy has recently been applied directly to the MTH reaction, revealing pronounced spatiotemporal temperature heterogeneities within individual HZSM-5 catalyst particles [65]. As the content of zeolite components increases, the thermal conductivity of the catalyst particles decreases, resulting in an increase in the temperature gradient within the catalyst particles (Fig. 6b and c). Reducing catalyst particle size shortens the diffusion path of molecules (Fig. 6d), reduces the temperature gradient, and makes the temperature distribution within the particles more uniform [65]. These measurements demonstrate that local temperature variations correlate strongly with zeolite density, particle size, and reaction progression, and directly influence the activation of hydrocarbon pool species and the utilization efficiency of Brønsted acid sites.

Taken together, these studies establish that temperature heterogeneity within zeolite catalyst particles is an intrinsic and dynamic feature of the MTH process. Rather than being a secondary effect, intraparticle temperature gradients actively affect local reaction rates, diffusion behavior, and catalyst utilization. Recognizing and quantifying this temperature nonuniformity represents a critical step toward a realistic description of spatiotemporal heterogeneity in MTH catalysis.

### Heterogeneity induced by pore connectivity

Pore connectivity within zeolite-based particles constitutes a critical yet often overlooked source of spatiotemporal heterogeneity. At the particle scale, the characteristic size of catalyst bodies already imposes strong constraints on pore accessibility and connectivity. Fluorescence nanoprobe imaging has revealed that as particle size increases, hierarchical pore networks become progressively less uniformly connected from the exterior to the core [59]. These size-dependent connectivity limitations translate directly into nonuniform diffusion fields, whereby reactants and intermediates preferentially reach peripheral regions while the particle interior experiences delayed or restricted mass transport, even when intrinsic zeolite microporosity remains unchanged.

**Figure 7.**
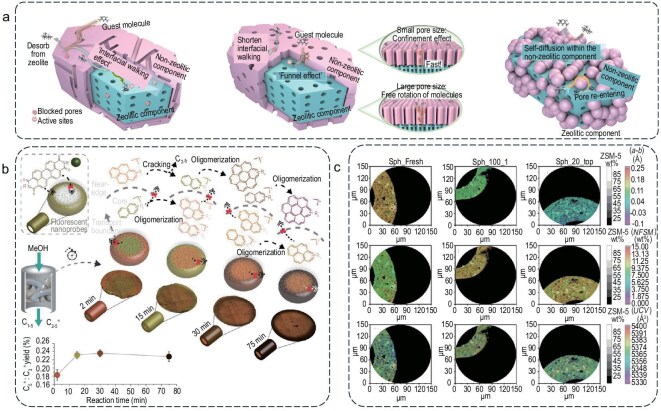
Heterogeneity induced by pore connectivity and coke distribution heterogeneity. (a) Schematic diffusion pathways of isooctane at zeolite–nonzeolite interfaces, showing poorly connected pores, well-oriented micro–mesopore alignment and random nonzeolitic component dispersion. Adapted from ref. [72] with permission of the American Chemical Society. (b) Schematic visualization of penetration depth and transport boundaries during MTH derived from ∼2 and 20 nm fluorescent nanoprobe accessibility in 3 mm Z:Kaolin catalyst bodies. Adapted from ref. [59] with permission of the Springer Nature. (c) Reconstructed powder X-ray diffraction computed tomography (PXRD-CT) slices of fresh (left), fully converted (middle), and 20%-converted (right) catalysts were refined, with the image contrast indicating the volume fractions of zeolite and alumina. Adapted from ref. [73] with permission of the Royal Society of Chemistry.

This heterogeneity is further amplified in multicomponent catalyst particles, where zeolitic and nonzeolitic domains coexist and interact through imperfectly connected pore networks. Combined gravimetric and infrared spectroscopy analyses demonstrated that, in such systems, global diffusion behavior differs markedly from the intrinsic diffusion within the zeolitic component, reflecting nonideal interconnectivity among microporous, mesoporous, and macroporous domains [71]. Poorly connected interfaces between zeolite crystals and surrounding binders or mesoporous phases act as diffusion bottlenecks, weakening the effective utilization of internal acid sites and promoting spatially nonuniform reaction rates across the particle (Fig. 7a). Recent model studies employing well-defined core–shell architectures have further established that pore orientation, spatial distribution of components, and pore sizes collectively govern interfacial diffusion efficiency between zeolitic and nonzeolitic domains [72]. When micropores and mesopores are well aligned and interconnected, molecular exchange across domains is facilitated. Conversely, misaligned or discontinuous pore networks induce pronounced transport heterogeneities that dominate overall catalytic behavior.

Taken together, these studies demonstrate that pore connectivity-induced heterogeneity emerges from a multiscale interplay between particle size and the complex architecture of multicomponent pore networks. Rather than being a mere geometric attribute, pore interconnectivity fundamentally controls the spatial distribution of molecular transport and reaction activity within catalyst particles.

### Coke distribution heterogeneity

In the MTH process, coke deposition at the particle scale is intrinsically heterogeneous, reflecting the coupled effects of transport limitations, local reaction environments, and multicomponent catalyst architectures. *Operando* and spatially resolved studies on zeolite-based catalyst bodies consistently showed that coke formation initiates preferentially within zeolite domains located near the external surface of the particle (Fig. 7b), where reactant accessibility and reaction rates are highest. As the reaction proceeds, coke species accumulate nonuniformly, generating pronounced radial gradients in coke content, with heavily coked outer regions and comparatively underutilized particle cores [59,73]. Such intraparticle coke gradients highlight that only a fraction of the catalyst body actively participates in the MTH reaction before diffusion limitations and deactivation set in.

Beyond its spatial distribution within zeolite domains, coke formation in shaped catalysts also involves a progressive redistribution of coke species from the zeolite component toward the surrounding matrix or binder phase (Fig. 7c). Advanced X-ray diffraction and absorption computed tomography revealed that part of the coke initially formed within zeolite micropores migrates into the alumina binder, leading to a more diffuse coke distribution at the particle scale [73–75]. This phenomenon was further supported by spectroscopic and microscopy studies showing that binder materials are not inert spectators but can host carbonaceous deposits, thereby acting as secondary sinks for coke species originally generated in the zeolite phase [75]. The extent of this coke transfer depends strongly on pore connectivity between zeolite and matrix domains, as well as on the chemical nature of the binder, which governs both adsorption strength and transport resistance for heavy hydrocarbons.

Taken together, these findings demonstrate that coke heterogeneity is not confined to individual zeolite crystals but evolves throughout the entire catalyst particle through coupled deposition and redistribution processes. The nonuniform accumulation of coke within zeolite domains, combined with its partial migration into the matrix phase, underscores the multiscale nature of deactivation in shaped catalysts and emphasizes the necessity of considering particle-scale coke dynamics when linking intrinsic zeolite chemistry to realistic MTH catalyst performance.

Particle-scale heterogeneity in MTH arises from the coupling of four factors. First, heterogeneity introduced during catalyst preparation and shaping creates nonuniform distributions of active domains within composite particles. Second, temperature distribution heterogeneity emerges from exothermic reaction heat release combined with limited heat transfer, leading to intraparticle thermal gradients and localized hot spots. Third, nonuniform pore connectivity and accessibility, particularly at zeolite–binder interfaces and within hierarchically structured particles, impose diffusion bottlenecks that generate pronounced radial gradients in reactant transport and active-site utilization. Finally, these structural and transport nonuniformities collectively lead to heterogeneous coke formation and migration, manifested as edge-to-core decreasing coke gradients and in composite catalysts, outward relocation of carbonaceous species toward binder matrices. Collectively, these four aspects demonstrate that particle-scale heterogeneity is a property of catalyst architecture, transport limitations, and reaction–deactivation coupling.

Importantly, the heterogeneities established at the particle scale are not confined within individual catalyst bodies. Instead, their cumulative effects propagate along the reactor, where interactions among particles, hydrodynamics, axial heat, and mass transfer give rise to bed-scale spatiotemporal heterogeneity. Understanding how particle-scale nonuniformities translate into reactor-scale gradients in temperature, reaction zones, and coke fronts is therefore essential, as discussed in the subsequent section.

## REACTOR-SCALE HETEROGENEITY IN THE MTH REACTION

Reactor-scale heterogeneity is an intrinsic and emergent feature of the MTH reaction, arising from the coupling of heat release, transport limitations, hydrocarbon-pool chemistry, and reactor hydrodynamics along the reaction coordinate. Strong exothermicity and diffusion constraints generate pronounced axial and radial temperature gradients, producing migrating thermal and reaction fronts that evolve as the catalyst deactivates. These thermal fields are intimately linked to spatial variations in hydrocarbon-pool composition, coke distribution, and product selectivity, giving rise to sharply differentiated reactivity zones across the bed. Moreover, reactor configuration—including contact time, flow regime, and feed type (MTO vs DTO (DME-to-oleffns))—further modulates these gradients and determines whether heterogeneity is exacerbated or suppressed. This section examines the formation, propagation, and mechanistic implications of bed-scale spatiotemporal nonuniformities.

### Temperature distribution heterogeneity

In fixed-bed reactors for the MTH process, temperature fields within the catalyst bed are intrinsically heterogeneous and evolve dynamically with reaction time as a direct consequence of the highly exothermic nature of methanol conversion. Both modeling and *operando* experimental studies demonstrated that, shortly after reaction initiation, localized temperature maxima emerge near the reactor inlet, where methanol conversion is most intense, giving rise to distinct hot zones that migrate along the bed as catalyst deactivation progresses [76,77]. As the active reaction front moves downstream over time, the axial temperature profile becomes strongly time-dependent rather than stationary, reflecting the coupled evolution of reaction kinetics, heat release, and catalyst deactivation.

**Figure 8.**
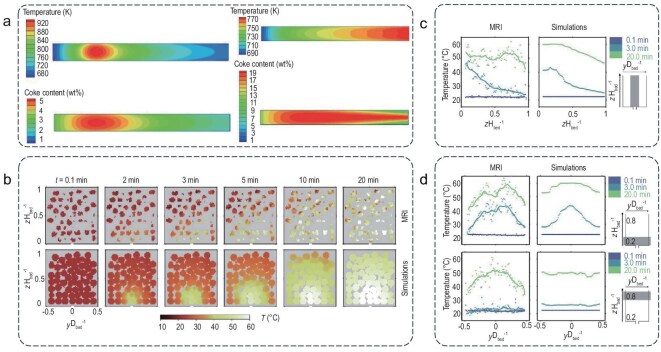
Reactor-scale temperature distribution heterogeneity. (a) Temperature and coke deposition distributions in the reactor at 500 s (left) and 3600 s (right). Adapted from ref. [77] with permission of the Elsevier. (b) Experimental (MRI, top) and simulated (bottom) temperature fields of a fixed bed during gas flow at 60°C and 100 L·min⁻^1^. Adapted from ref. [78] with permission of the Elsevier. (c) Averaged axial temperature profiles obtained from MRI measurements and numerical simulations at three time points (0.1, 3, and 20 min) during heating experiments. Adapted from ref. [78] with permission of the Elsevier. (d) Averaged radial temperature profiles obtained from MRI measurements and numerical simulations at *z*·*H*_bed_^−1^ = 0.2 and *z*·*H*_bed_^−1^ = 0.8 for three time points (0.1, 3, and 20 min) during heating experiments. Adapted from ref. [78] with permission of the Elsevier.

Beyond this temporal evolution, fixed-bed temperature heterogeneity also manifests pronounced directional differences. Along the axial direction, *in situ* luminescence thermometry and magnetic resonance imaging (MRI) revealed clear temperature gradients from the inlet to the outlet of the bed, with sequential temperature maxima appearing at different axial positions as the reaction zone propagates [76,78]. In contrast, radial temperature gradients arise from nonuniform heat removal at the reactor wall and heterogeneous gas–solid heat transfer within the packed bed. Computational fluid dynamics (CFD) simulations of MTH fixed-bed reactors consistently predicted higher temperatures in the bed core relative to the wall (Fig. 8a), particularly under high space velocities or limited external heat transfer, leading to asymmetric radial temperature distributions superimposed on the axial gradients [77]. Experimental MRI-based thermometry further confirmed that axial and radial temperature gradients coexist and jointly define a fully three-dimensional thermal landscape within fixed beds (Fig. 8b–d), even under nominally steady operating conditions [78].

Taken together, these studies establish that temperature heterogeneity in MTH fixed-bed reactors is inherently spatiotemporal, evolving with reaction time and exhibiting distinct axial and radial characteristics.

### Reactant and intermediate distribution heterogeneity

In fixed-bed reactors for the MTH process, the distribution of reactants and intermediate species along the catalyst bed is inherently nonuniform, reflecting the strong coupling among mass transport, reaction kinetics, and catalyst deactivation. At the reactant distribution level, methanol and DME exhibit different axial concentration profiles. Spatiotemporal analyses showed that methanol is preferentially consumed near the reactor inlet, leading to a sharp reaction front and pronounced reactant depletion in the downstream sections, whereas more diffusion-limited DME displays an extended penetration depth along the bed, effectively elongating the reaction zone and moderating local reactant enrichment [7]. This contrast highlights that even chemically similar C1 reactants can form distinct spatial reaction regimes due to differences in diffusion and surface barriers.

As the reaction proceeds, the nonuniform distribution of reactants directly translates into heterogeneous formation of hydrocarbon pool species, which serve as key autocatalytic intermediates in MTH chemistry. Solid-state NMR and bed-segmented analyses over HZSM-5 demonstrated that reactive carbocations and light alkenes are predominantly generated and maintained in the upper catalyst layers, where methanol conversion is highest, while heavier methylbenzenes accumulate preferentially near the inlet and show diminished reactivity further downstream [79]. This axial segregation of hydrocarbon pool species implies that distinct reaction cycles coexist at different bed positions, with olefin-based pathways remaining active over a broader section of the bed, whereas aromatic-based cycles are spatially confined to regions of high reactant chemical potential.

In parallel, the distribution of volatile species further reinforces this spatiotemporal heterogeneity. Light olefins and other volatile hydrocarbons formed in the upstream readily desorb and migrate downstream, sustaining secondary reactions and partial catalyst utilization beyond the primary reaction front. However, in SAPO-34-containing fixed beds, *operando* observations revealed that rapid coke formation near the inlet progressively suppresses the local release and transport of volatile intermediates, resulting in a shrinking effective reaction zone and accelerated deactivation of upstream catalyst layers [80]. Consequently, the interplay between volatile product transport and localized deactivation governs the dynamic redistribution of reaction activity along the bed.

These studies demonstrate that the MTH fixed-bed reactor operates far from spatial uniformity. Reactant depletion, intermediate accumulation, and volatile species transport are all strongly position-dependent and evolve with time on stream. Such intrinsic heterogeneity underscores the necessity of explicitly considering spatiotemporal distributions of reactants and intermediates when linking molecular-scale MTH chemistry to catalyst-bed performance and reactor-scale behavior.

### Coke distribution heterogeneity

**Figure 9.**
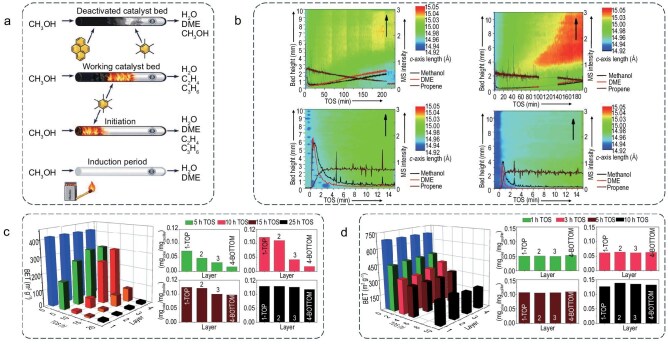
Reactor-scale coke distribution heterogeneity. (a) Schematic illustration of the time evolution of a catalyst bed during an MTO experiment described by the ‘cigar-burn’ model. Adapted from ref. [81] with permission of the Springer Nature. (b) The evolution of the *c*-axis structural parameters of a fixed-bed catalyst during the MTO reaction was investigated *operando* using fast high-energy X-ray diffraction imaging. Shown are the experimental results for SAPO-34 zeolite with a silicon content of 4% at a flow rate of 50 mL·min⁻^1^ (top), together with a magnified view of the first 15 min of reaction (bottom), where the arrows indicate the direction of reactant flow. Adapted from ref. [83] with permission of the Wiley-VCH GmbH. (c) At 400 °C, the BET area on partially deactivated catalyst layers of ZSM-5 (left) and the normalized amounts of total coke in the different fractions of ZSM-5 (right) after reaction. Adapted from ref. [88] with permission of the Royal Society of Chemistry. (d) At 400 °C, the BET area on partially deactivated catalyst layers of β-CP-806 (left) and the normalized amounts of total coke in the different fractions of β-CP-806 (right) after reaction. Adapted from ref. [88] with permission of the Royal Society of Chemistry.

In fixed-bed reactors for the MTH process, coke formation exhibits pronounced spatiotemporal heterogeneity along the catalyst bed, a phenomenon classically conceptualized by the ‘cigar-burn’ model (Fig. 9a). In this framework, catalyst deactivation proceeds as a moving front, where coke preferentially accumulates near the reactor inlet and progressively propagates downstream with time on stream, leaving behind a deactivated zone and maintaining an active methanol conversion region [81,82]. This axial heterogeneity reflects the autocatalytic nature of MTH chemistry, in which methanol-rich upstream zones promote rapid formation of hydrocarbon-pool species and their subsequent transformation into coke. Time- and space-resolved high-energy *operando* X-ray diffraction has provided direct structural evidence for this dynamic behavior. Importantly, Wragg *et al.* [83] demonstrated that the direction of deactivation propagation depends critically on contact time (Fig. 9b). This reversal highlights that coke deposition is not solely governed by inlet reactant concentration, but by the coupled balance between conversion rate, residence time, and product reactivity along the bed. Under short contact time (high space velocity), the upper part of the bed becomes deactivated first, which is due to the rapid conversion of methanol and the accumulation of coke deposition. Subsequently, the deactivation front advances downstream. In contrast, under long contact time (low space velocity), the lower part of the bed becomes deactivated earlier and the deactivation front gradually expands upstream [83]. The chemical properties of coke deposited on different axial positions of the fixed bed are also different. Kinetic model studies on the combustion of coke indicated that the easily reactive coke is mainly distributed in the inlet area of the reactor, while the refractory coke is distributed throughout the bed [84].

*Operando* spectroscopic and diffraction-based imaging techniques have directly visualized this nonuniform coke distribution. Time- and space-resolved UV–vis, UV–Raman, and high-energy X-ray diffraction studies consistently revealed steep axial gradients in coke content, with the highest concentration localized near the inlet and diminishing toward the outlet during early reaction stages [85,86]. As the reaction progresses, this coke front migrates through the bed, confirming that deactivation is governed by spatially localized coke deposition rather than uniform aging of the catalyst. Importantly, the position and sharpness of the coke front are sensitive to operating conditions, among which gas flow rate plays a crucial role. Higher space velocities tend to broaden the reaction zone and moderate coke accumulation at the inlet, whereas lower flow rates prolong local residence times, accelerating coke growth and sharpening axial gradients [82].

Beyond spatial distribution, the nature of coke species also differs across different axial positions of the bed. In the initial stage of reaction, the coke deposits at the top of the bed directly originate from the reactants. However, the coke deposits at the middle and bottom of the bed are formed through the secondary reaction of MTO products (ethylene and propylene) during their downward diffusion process [84,85]. Such differentiation underscores that coke heterogeneity in fixed beds is both spatial and chemical in nature. Comparisons among different pore topologies further highlight this effect. In medium-pore zeolites such as H-ZSM-5 and H-ZSM-22, coke formation is strongly coupled to channel orientation and accessibility, yet the underlying mechanisms are fundamentally distinct. In H-ZSM-5, three-dimensionally interconnected straight and sinusoidal channels enable coke species to nucleate at channel intersections and propagate along multiple diffusion pathways, leading to a more distributed but anisotropic coke accumulation throughout the crystal. In contrast, H-ZSM-22, characterized by one-dimensional TON channels accessible only from crystal ends, exhibits coke growth that is highly localized near channel mouths, with rapid pore blocking and limited penetration into the crystal interior, resulting in sharper spatial gradients along the bed and faster local deactivation [85,87]. A further contrast can be drawn between H-ZSM-5 and Beta zeolite, which both possess three-dimensionally connected pore systems but differ substantially in channel size and intersection geometry (Fig. 9c and d). While the medium-pore MFI framework of H-ZSM-5 constrains the growth and migration of coke precursors within intersecting 10-MR channels, leading to spatially anisotropic yet relatively confined coke deposition, the larger-pore Beta zeolite facilitates faster diffusion and interconversion of bulky hydrocarbon-pool species. Consequently, coke formation in Beta is more spatially dispersed along the fixed bed, with less sharply defined axial fronts but a higher tendency toward the formation of heavier, more condensed polyaromatic coke at earlier stages, ultimately accelerating global deactivation despite weaker local pore blocking [85,88].

Finally, reactor configuration exerts a decisive influence on coke heterogeneity, particularly for SAPO-34 catalysts. In fixed-bed reactors, SAPO-34 displays strong axial gradients in coke deposition consistent with the cigar-burn behavior, whereas in fluidized-bed reactors the continuous mixing of catalyst particles leads to a much more uniform spatial coke distribution at any given time on stream [82,89]. These contrasts demonstrate that the observed coke patterns are not solely intrinsic to the zeolite but emerge from the interplay between catalyst topology and reactor hydrodynamics.

**Figure 10.**
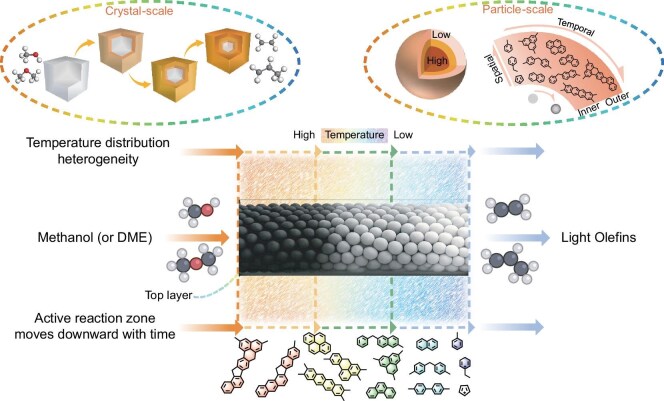
Summary of spatiotemporal heterogeneity in the MTH reaction.

Reactor-scale studies show that MTH is governed by moving reaction–diffusion–deactivation fronts, not ideal plug-flow behavior (Fig. 10). Along the fixed bed, intense exothermicity and mass-transfer limitations generate pronounced axial and radial temperature gradients, with hot spots and thermal fronts that migrate as the catalyst deactivates, as revealed by CFD modeling, luminescence thermometry, and MRI. These thermal nonuniformities are tightly coupled to heterogeneous distributions of hydrocarbon-pool species and products. Aromatics and heavy intermediates accumulate preferentially near the inlet, while their composition and reactivity evolve layer-by-layer down the bed, producing position-dependent selectivity and deactivation kinetics. In parallel, coke deposition forms highly structured axial ‘cigar-burn’ fronts and radial core–shell patterns, whose propagation rate and direction are controlled by contact time, reactor hydrodynamics, zeolite topology, and feed (MTO vs DTO), leading to sharp contrasts between strongly graded fixed beds and more homogeneous semifluidized/fluidized systems. Together, these *operando* and multiscale insights establish that bed-scale MTH heterogeneity arises from the dynamic interplay of heat release, molecular transport, hydrocarbon-pool chemistry, and reactor flow regime. Rational process and catalyst design must explicitly manage these spatiotemporal gradients to stabilize olefin selectivity and prolong catalyst lifetime.

## CONCLUSIONS AND OUTLOOK

Taking the MTH reaction as a prototype, this review employs multiscale analysis to elucidate the spatiotemporal heterogeneity in catalytic processes, offering insights for other zeolite‑catalyzed processes including FCC, hydrocarbon cracking, CO₂ hydrogenation, syngas conversion, etc.

In this review, we systematically dissect that spatiotemporal heterogeneity in the MTH reaction exhibits across four levels: (i) the molecular diffusion scale, where intrinsic molecular properties, zeolite topology, acid site microenvironments, surface barriers, confinement-induced anomalous transport effects, etc., give rise to pronounced anisotropic and dynamic transport heterogeneity; (ii) the crystal scale, where synthesis-induced compositional zoning, temperature-dependent reaction–diffusion coupling, and topology-sensitive coke deposition lead to spatially heterogeneous activity and deactivation within crystals; (iii) the particle scale, where preparation parameters, intraparticle temperature gradients, pore connectivity, and binder interactions lead to localized heterogeneous reaction microenvironments and thus segregated coke deposition; and (iv) the reactor scale, where the coupling of endo/exothermicity, mass/heat transport, and hydrodynamics generates moving thermal and coke fronts, resulting in axial and radial gradients in temperature, intermediate distribution, and deactivation. Collectively, zeolite-catalyzed MTH reactions inherently exhibit multiscale heterogeneities, governed by the complex interplay among intrinsic material properties, transport limitations, and reactor engineering.

Spatiotemporal heterogeneity severely limits zeolite-catalyzed large-molecule conversions such as MTA, aromatics conversion, and FCC reactions. Confined diffusion leads to reactant/intermediate accumulation at pore mouths or near-surface region, accelerating localized coke formation and rapid deactivation, while leaving internal active sites underutilized [90,91]. To improve molecular diffusion and acid site accessibility, Baolian Su’s team developed a bio-inspired hierarchical pore architecture, based on which they proposed Su’s Law (generalized Murray’s Law) [90,92–94]. This law guides the precise construction of interconnected macro-meso-microporous structures in single zeolite crystals [95,96], promoting material research from ‘trial-and-error’ to demand-oriented rational synthesis [96]. Building on Su’s Law, they established a ‘Pore Science and Engineering’ framework integrating pore chemistry and structure design [90,92,94–96]. This provides theoretical support for rational pore design, aiding molecular diffusion and high-performance applications, and offers a basis for alleviating spatiotemporal heterogeneity in zeolite-catalyzed reactions via hierarchical pores. Beyond hierarchical zeolites, extra-large pore zeolites offer another route to enhance mass transfer for macromolecules. Recent advances in structure-directing agents have enabled the synthesis of extra-large pore zeolites (pore apertures >12-MR), crucial for heavy oil conversion [97,98]. For instance, ZMQ-1 (28-MR, 22.76 × 11.83 Å), NJU120-1 (22-MR, 15.49 × 12.34 Å), and ZEO-5 (20-MR, 14.3 × 13.5 Å) shorten diffusion paths and improve active site accessibility, delivering catalytic cracking performance superior to commercial USY zeolite [97,98]. Thus, hierarchical and extra-large pore zeolites represent key strategies to overcome diffusion barriers in conventional microporous materials, reducing spatiotemporal heterogeneity and boosting macromolecular reaction efficiency.

In contrast, for small molecule reactions such as MTO and direct conversion of CO_2_ or syngas over bifunctional catalysts (composite of oxide and zeolite), spatiotemporal heterogeneity on zeolite serves as a double-edged sword effect. While excessive heterogeneity can impede mass transport and accelerate deactivation, a controlled degree of nonuniformity can steer reaction pathways and enhance targeted product selectivity. For instance, pre-coking and spatially presituated ‘coke’ species within SAPO-34 crystals have been shown to redistribute reaction zones, promote the formation of aromatic-cycle intermediates, and substantially enhance ethene selectivity without apparently damaging catalyst lifetime [99]. Similarly, directed steam‑cracking of coke into active naphthalenic intermediates, which are predominantly located in the central region of the crystals can rapidly restore catalytic activity while simultaneously increasing the selectivity of ethene and propylene to 85% [100]. The chemical basis for selectivity regulation through controlled heterogeneity lies in the interplay between diffusion, reaction, and coking. These findings underscore that, achieving spatiotemporal coupling between diffusion and reaction is crucial for optimizing catalyst design in zeolite-catalyzed small-molecule transformations.

Although spatiotemporal heterogeneity is now widely recognized as a defining feature of zeolite-catalyzed reactions, its direct experimental interrogation remains highly challenging. CFM, structured illumination microscopy, and APT techniques are limited by their spatial resolution (with submicrometer and subnanometer resolution), thus only providing ensemble-averaged observations and lacking the capability for molecular- or even atomic-level imaging. Noncontact atomic force microscopy can realize atomic-level resolution, yet it cannot spatially resolve molecular species within catalyst crystals or particles [57]. Collectively, most techniques can offer high spatial resolution but lack dynamic, *operando* capability and precise molecular structure identification, or can provide detailed molecular structure information but lack temporal resolution ability. Bridging this gap will require the development of novel techniques that integrate high-resolution spatial mapping with molecular or even atomic-scale imaging.
